# Therapeutic approaches in pelvic bleeding of neoplastic origin

**Published:** 2014-09-25

**Authors:** LR Popovici, A Ciulcu, B Dorobat, M Dumitraşcu, VV Horhoianu, M Cirstoiu

**Affiliations:** * University Emergency Hospital, Bucharest; ** Dr. I. Cantacuzino Clinical Hospital

**Keywords:** pelvic haemorrhage (pelvic bleeding), hypogastric artery ligation, uterine artery embolization, Mohs’ paste

## Abstract

Abstract

Objectives: The aim of this study is to present the main – surgical and non-surgical – therapeutic approaches (or methods) used in the treatment of pelvic bleeding of neoplastic origin.

Materials and methods: analysis of the materials found in the literature on this subject.

Results: Among the surgical methods used, hypogastric artery ligation is the oldest therapeutic approach in cervical bleedings of neoplastic origin. Due to the frequent recurrence of haemorrhages, mere ligation has been proven not to be sufficient, but necessitating the concomitant ligation of the lumbo-ovarian, round and uterosacral ligaments. In the case of severe bleedings, difficult to control, direct embolization of the hypogastric artery below the level of ligation is usually practiced. As for the non-surgical methods used, we chose to present uterine artery embolization and the application of haemostatic Mohs’ paste. Uterine artery embolization consists in the permanent occlusion of the uterine arteries in neoplastic abundant haemorrhages, when the intervention includes the use of permanent embolic material. Stopping the bleeding within the first 24 hours from the embolization means that the intervention has been a success, and provides the patient with the possibility to continue the therapy protocol according to her stadialization. Recent studies of the Japanese researchers have indicated the possibility to use the Mohs’ paste for haemostatic purposes on patients with cervical bleedings of medium intensity, in cases of advanced cervical cancer.

Conclusions: With severe haemorrhages – occurring spontaneously or during surgery – the emergency haemostatic intervention consists in the bilateral hypogastric artery ligation. With long-lasting haemorrhages of medium intensity, we usually resort to uterine artery embolization, since this is a minimally invasive method and may also be performed with abundant bleeding under emergency pressure. The application of the Mohs’ paste for haemostatic purposes is a new therapeutic method, whose efficiency cannot be yet estimated.

## Introduction

In severe complications of cervical cancer, utero-vaginal bleeding occupies a significant place of high priority, if we consider the urge to stop it.

 Such local bleedings generally appear in advanced stages (III and IV) of cancer evolution. 

They raise a serious question concerning the medical approach – as they are difficult to control, cause anaemia to the patient and exclude any possibility for the patient to undergo endocavitary radiation therapy.

 Current therapeutic approaches

 To this purpose, we shall review the therapeutic approaches (or methods) that are available at present, and which could be used according to the relevant endowment of the clinical unit, as well as to the local and general status of the patient. 

A. Surgical approaches:

 a. hypogastric artery ligation

 B. Non-surgical approaches:

 a. uterine artery embolization 

 b. local application of haemostatic Mohs’ paste

 A. Surgical methods

 a. Hypogastric artery ligation

 Until three decades ago this used to be the only possibility to control – that is, stop – cervical bleedings of neoplastic origin. 

 When they could not be stopped by the patient’s undergoing a medical treatment or by means of small surgery (haemostatically, by tight vaginal plugging with peroxide-treated pads), we used to resort to intrapelvic vascular ligation [**3**].

 In 1893, Howard Kelly performed the bilateral hypogastric artery ligation (in premiere) at John Hopkins Hospital, in order to control a haemorrhage started during the performance of a hysterectomy in a case of uterine cancer.

 Later on, this kind of intervention was introduced in the general practice by Mengert and further developed by Burehell; the latter proving that, should both hypogastric arteries have been ligated, then the blood pressure was decreased in 85% of the cases – in the area in question.

 Since mere ligation was not sufficient (as bleeding used to reappear), it was recommended to proceed to a concomitant ligation of the lumbo-ovarian, round and uterosacral ligaments.

 What should also be mentioned is a technical detail: in order to avoid the injury of the hypogastric vein (located under the artery), the sheath (adventitia) of the artery may be incised and then lifted up, so that the Deschamps needle may be introduced through the tunnel created: the ligation is performed with a thick thread – not to cut the artery [**3**].

**Fig. 1:**
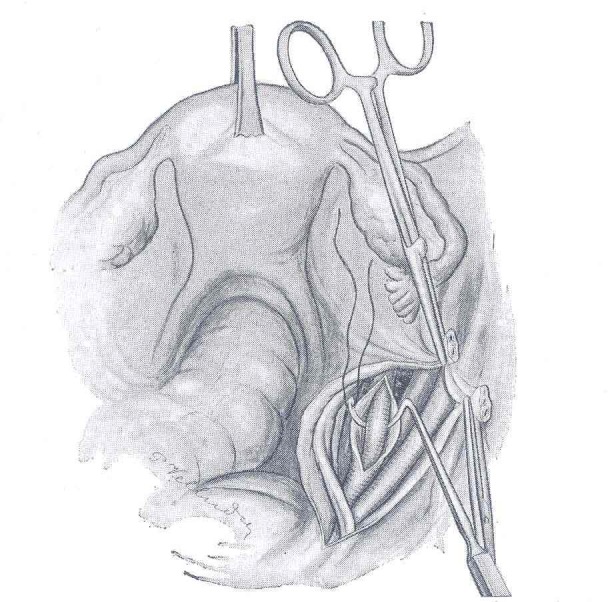
Technique of ligation of the right hypogastric artery (after Proust and Maurer). The Deschamps needle is introduced between the hypogastric artery and the adventitia of the blood vessel, which was first incised; this way the vein is protected

 There are also authors (like Burchell) who performed the double ligation of the hypogastric artery (in order to avoid repermeabilization) [**2**].

 The reduction of blood in the relevant area allows the achievement of thrombosis in the respective bleeding blood vessels (Burchell).

 With severe pelvic haemorrhages that are difficult to control (due to multiple bleeding sources), Saueracker practiced hypogastric artery embolization (through one side – or both sides – according to the angiography), by direct injection of an embolic material into the artery – below the level of ligation, on the hypogastric artery.

**Fig. 2:**
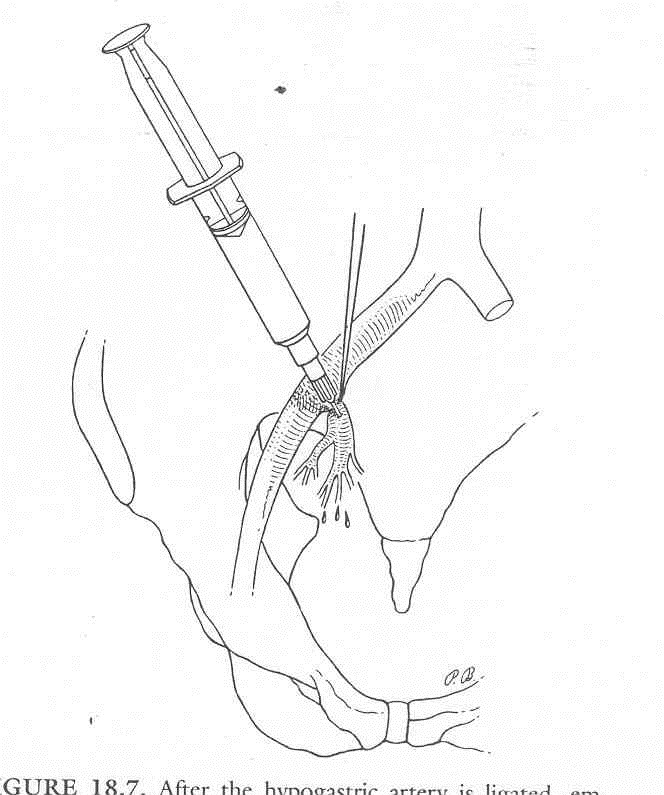
After hypogastric artery ligation, the embolization of the bleeding blood vessels may be achieved through direct injection of the embolic agent into the artery – below the level of ligation

 Conclusions: Hypogastric artery ligation is the most conventional surgical procedure that can control severe pelvic bleeding in cases of advanced cervical cancer with tumour block, should a radical surgical intervention not be possible to be performed (in stages overdue for surgery).

 B. Non-surgical methods

 a. Uterine artery embolization 

 It was introduced as a therapeutic method during the 1960s.

 Of the doctors who used this approach in the management of severe bleeding in advanced stages of uterine cancer, the following should be mentioned: Harima, Pisco, Anthanasoulis and many others [**2**].

 In the case of old patients or patients who presented related (cardio-pulmonary) organ defects, and considering the precarious and local hemodynamic status of the same – which excluded any possibility for a surgical intervention – the only therapeutic option left was embolization.

 This method consisted in the final occlusion of some end arteries – respectively of the uterine arteries. 

 Technical issues

 Intravascular embolization is performed by an interventional radiologist – under angiographic control.

 A varied range of materials are used in embolization, in order to achieve a temporary or permanent vascular occlusion. 

 Of the resolvent embolic agents, the following should be mentioned: Tachocomb, Gelaspon, Gelfoam, etc.; of the non-resolvent ones: PVA (polyvinyl alcohol particles) – respectively Contour, Ivalon, etc.

 Embospheres are part of the second category (respectively, used for permanent occlusion), but they raise the inconvenience of having a high price. 

 The place of bleeding is identified by angiographic means; the brachial artery is catheterized under local anaesthesia at the level of the fringe (plica) of the elbow joint, by using the Seldinger technique – a method that allows retrogressive access to the hypogastric artery and its branches (the uterine arteries). One advantage in the brachial approach is that an endovascular puncture is performed unilaterally in order to embolize the right and left uterine arteries. 

**Fig. 3:**
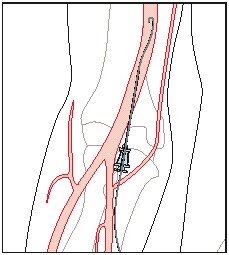
Catheterization of the brachial artery

 The embolic agents – mixed up with the contrast substance (for visualization purposes) are injected through the catheter; the surgery takes place at the level of both uterine arteries.

 After performing the control angiography, at the end of the intervention, the catheter is withdrawn [**1**].

 Pain control may be achieved with the help of Midazolam, Tramadol or Remifentanil [**5**].

 To stop the haemorrhage within the first 24 hours from embolization means a success in such interventions and provides a possibility for the patient to continue the therapy protocol according to her relevant stage of cancer. 

 We further present two suggestive images taken before and after embolization on a 35-year-old female patient – diagnosed with "radiated exocolic neoplasm", stage III [**6**]; what should be noticed in the second post-embolization image is the absence of the contrast substance at the level of the uterine structures.

**Fig. 4:**
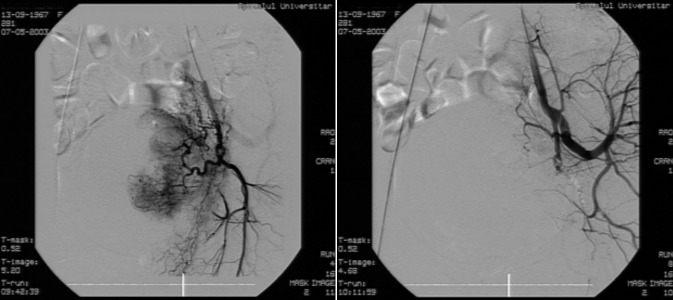
35-year-old female, radiated exocolic neoplasm, stage III, metrorrhagia – presentation before and after embolization

 In special conditions, when the brachial approach cannot be performed, we opted for the femoral approach. 

**Fig. 5:**
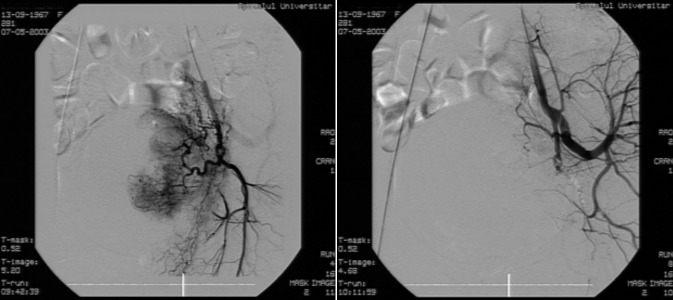
Fig. 5 Catheterization of the femoral artery – under local anaesthesia – allows retrogressive access to the hypogastric artery and its branches

 The femoral artery was first of all found and, after a local anaesthesia with 1% of lidocaine (Xilina 1%), the artery was punctured at about 2-3 cm below the line joining the anterior superior iliac spine and the pubis. At the very moment the arterial, pulsatile blood squirt was detected, the flexible guide wire of the short guiding catheter was introduced in the needle and, subject to radiological control; the easy ascension of the same was followed through the common femoral artery and the external iliac artery toward the abdominal aorta, on a distance of about 15-20 cm.

 While the puncture needle was withdrawn, the place of puncture was compressed not to bleed out, and the guide wire was left behind. Then the artery’s adventitial sheath was introduced on the guide wire, until the guide wire passed by the proximal end of the adventitial sheath with minimum 2 cm. The adventitial sheath was introduced into the artery, by following the withdrawal of the short guide wire.

 b. Local application of the haemostatic Mohs’ paste

 In 1930, Frederic F Mohs imagined an original method for the chemical fixation (solidification) of skin tumours, by using 20% of zinc chloride (Mohs’ paste), which would cause necrosis of the tissue, but preserve (maintain) the tissular microscopic structures. 

 He then developed a technique of tumour resection in series by using a chemical-surgical technique. 

 The Japanese researchers have recently presented the possibility to use the Mohs’ paste – for haemostatic purposes – with patients suffering from cervical bleeding (in advanced cervical cancer) [**4**].

## Case description

In April 2012, a multiparous female, aged 55 was hospitalized in conditions of medical emergency in the University Hospital of Kagoshima – she was reported to have undergone a massive vaginal haemorrhage due to advanced cervical cancer. One year before, she had been diagnosed with FIGO stage III B, and histopathologically, because she was suffering from squamous-cell cervical carcinoma.

 After that, she underwent external and internal radiation, followed by chemotherapy with Cisplatin and Irinotecan.

 Upon hospitalization the patient was in hypovolemic shock (the amount of lost blood was estimated at 1300 ml or more); TA=78/42 AV -120/1’ Hb=5.4 g/l; she received 8 units of red cell mass.

 The clinical exam reported the fragility of the patient’s cervical tumour and the entire infiltration of the vaginal wall with blood.

 Approach

 12 hours from hospitalization, the Mohs’ paste was directly applied on the most active area of the tumour bleeding by using compresses (or tampons) – for a few minutes – at reduced pressure.

 Every 24 hours, the compress would be replaced; after 10 days of application, the bleeding area of the tumour was removed; nevertheless, the patient died due to multiple organ failure, but not because of vaginal bleeding.

 Composition: mixture of zinc chloride (50gr), distilled water (25ml), zinc iodide – starch solution (19g) and glycerol (15ml).

 The haemostatic effect of the paste was explained by the authors by the necrotic action of the mixture of zinc to the newly formed blood vessels, and by the increase of blood thickness due to the action of glycerol.

**Fig. 6:**
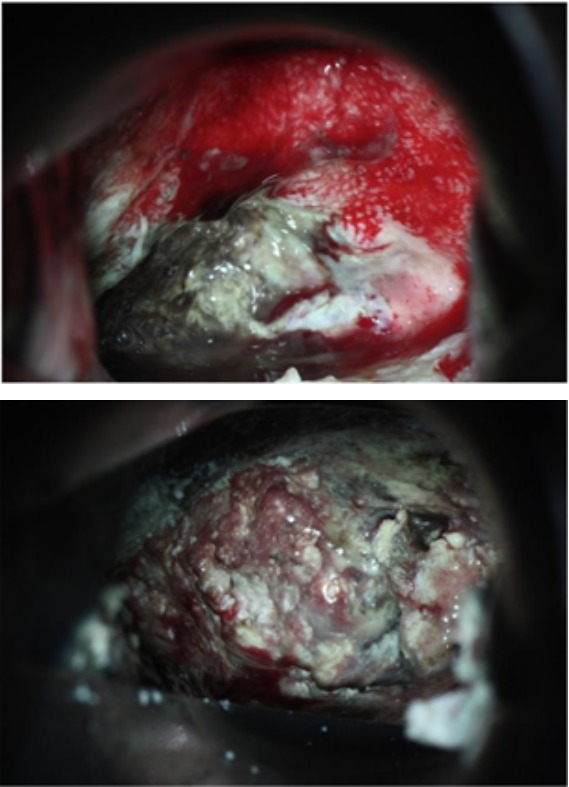
Images before and after applying the Mohs’ paste

This case presentation of the Japanese authors is truly remarkable, but it cannot be used unless we treat the long-lasting haemorrhages of medium intensity (this case was presented in Shintaro Yanazume, Haruhiko D – "New hemostatic method using Mohs’ paste for fetal genital bleeding in advanced cervical cancer").

## Conclusion 

With severe bleeding – occurring spontaneously or during surgery – the emergency haemostatic intervention will be the bilateral hypogastric artery ligation. In order to avoid repermeabilization, the ligation may be doubled and, should we want to increase the efficacy of the haemostasis, we shall use uterine artery embolization by injecting the material directly below the level of ligation (as presented above).

 With long-lasting bleeding of medium intensity, we should resort to uterine artery embolization; this minimally invasive method may also be approached in the case of significant bleeding in cases of emergency. The method of intervention will depend on the cooperation between the gynaecologic surgeon, the oncologist and the interventional radiologist. 

The application of the Mohs’ paste – for haemostatic purposes – makes a new acquisition and cannot be therefore fairly estimated; we still need a larger number of cases to be able to say, as a definite conclusion, that it proposes an efficient method to stop the bleeding on a long run; anyway, this technique shall be applied to cases of long-lasting bleeding. 

 As temporary methods used to stop the bleeding, we could also use haemostatic drugs like Tachosil, Tachocomb or other types of haemostatic powders until the final intervention meant to stop the haemorrhage (uterine artery embolization).
